# Evaluation of the Binding Mechanism of Human Defensin 5 in a Bacterial Membrane: A Simulation Study

**DOI:** 10.3390/ijms222212401

**Published:** 2021-11-17

**Authors:** Tadsanee Awang, Phoom Chairatana, Ranjit Vijayan, Prapasiri Pongprayoon

**Affiliations:** 1 Department of Chemistry, Faculty of Science, Kasetsart University, Bangkok 10900, Thailand; tadsanee.a@ku.th; 2 Department of Microbiology, Faculty of Medicine Siriraj Hospital, Mahidol University, Bangkok 10700, Thailand; phoom.chairat@gmail.com; 3 Department of Biology, College of Science, United Arab Emirates University, Al Ain P.O. Box 15551, United Arab Emirates; 4 Big Data Analytics Center, United Arab Emirates University, Al Ain P.O. Box 15551, United Arab Emirates; 5 Zayed Center for Health Sciences, United Arab Emirates University, Al Ain P.O. Box 17666, United Arab Emirates; 6 Center for Advanced Studies in Nanotechnology for Chemical, Food and Agricultural Industries, KU Institute for Advanced Studies, Kasetsart University, Bangkok 10900, Thailand

**Keywords:** antimicrobial peptides, human defensin 5, lipopolysaccharide, molecular dynamics simulations

## Abstract

Human α-defensin 5 (HD5) is a host-defense peptide exhibiting broad-spectrum antimicrobial activity. The lipopolysaccharide (LPS) layer on the Gram-negative bacterial membrane acts as a barrier to HD5 insertion. Therefore, the pore formation and binding mechanism remain unclear. Here, the binding mechanisms at five positions along the bacterial membrane axis were investigated using Molecular Dynamics. (MD) simulations. We found that HD5 initially placed at positions 1 to 3 moved up to the surface, while HD5 positioned at 4 and 5 remained within the membrane interacting with the middle and inner leaflet of the membrane, respectively. The arginines were key components for tighter binding with 3-deoxy-d-manno-octulosonic acid (KDO), phosphates of the outer and inner leaflets. KDO appeared to retard the HD5 penetration.

## 1. Introduction

**Figure 1:**
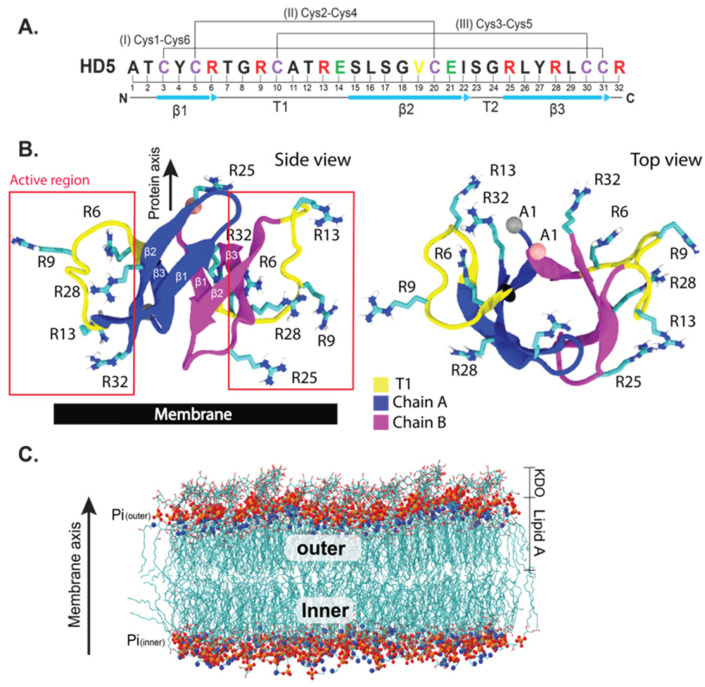
(**A**) Amino acid sequences of HD5. Conserved cysteines are shown in purple. Valine, arginine, and glutamic acid are shown in yellow, red, and green, respectively. The secondary structure is also indicated. (**B**) Cartoon representation of a dimeric HD5. The arginine-rich active region is located within the red box. The lipopolysaccharide (LPS) membrane used in this work is shown in (**C**). Only 3-deoxy-*d*-manno-octulosonic acid (KDO) and lipid A are involved in this LPS membrane.

The emergence of antibiotic-resistant bacteria in both hospital and community settings has become a significant problem worldwide. Moreover, the paucity of more effective antibiotics in the drug pipeline is further worsening this issue [1,2]. Therefore, it is necessary to develop novel therapeutic strategies to treat patients who get infected with antibiotic-resistant pathogens. The mechanism of the host innate immune system has the potential to be explored for developing new drugs. The innate immune system provides a first-line defense against microbial invaders by using several mechanisms, including the release of antimicrobial peptides (AMPs) [3]. AMPs are essential effectors of the innate immune system, and they typically possess broad-spectrum antimicrobial activities [4]. In humans, there are two major types of AMPs, namely defensins [5] and the cathelicidin LL-37 [6]. Defensins are cationic amphipathic peptides that contain an arginine-rich region called “the active region” on one side and a hydrophobic patch on the other side (Figure 1A,B). The cationicity of defensins mediate their bacteria-killing activities [7,8,9]. In addition, defensins are cysteine-rich peptides; therefore, they are divided into three subfamilies—β-defensins, α-defensins, and θ-defensins—depending on the regiospecific disulfide-bond linkages [10]. Both α- and β-defensins share similar β-sheet core structures (β1–β3) with three conserved disulfide bonds (Cys1—Cys6, Cys2—Cys4, and Cys3—Cys5 in α-defensins; and Cys1—Cys5, Cys2—Cys4, and Cys3—Cys6 in β-defensins) (Figure 1A), while θ-defensins form cyclic structures [10]. Six α-defensins have been identified in humans, which are neutrophil defensins (human neutrophil defensins 1–4 (HNP1–HNP4)) and enteric defensins (human defensins 5 (HD5) and 6 (HD6)) [7]. HNP1–HNP4 are found in neutrophils, while HD5 and HD6 are expressed in Paneth cells of the small intestine [11]. Human defensins were found to inactivate bacteria by disrupting and permeating bacterial membranes [8,12]. The active region was reported to perturb the orientation of lipid headgroups after binding to them [12,13]. However, the microscopic details of membrane binding and insertion remains poorly understood. Among the six α-defensins, HD5 shows high killing activities against Gram-negative and Gram-positive bacteria [14]; however, the detailed mechanism of how HD5 disrupts the bacterial cell membrane remains unclear. 

**Figure 2:**
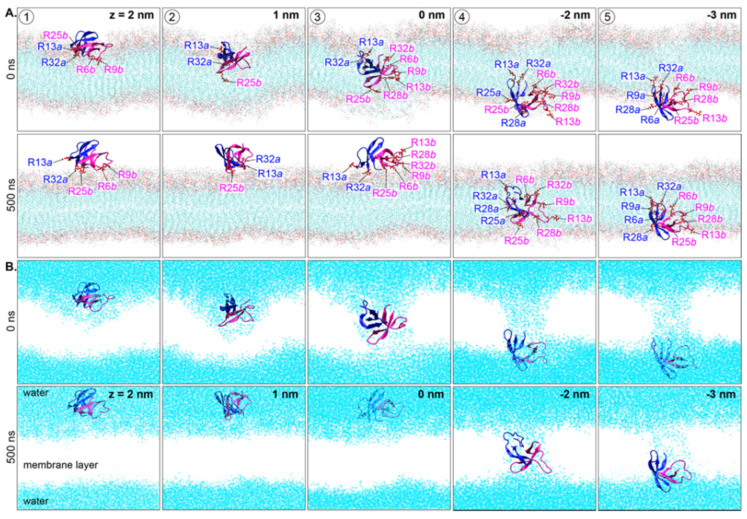
(**A**) Starting and final orientations of dimeric HD5 in a membrane at five positions. Water molecules in each system are shown in cyan in (**B**). Chains *a* and *b* of HD5 dimer are displayed in blue and pink. Key arginine (R) residues that are in contact with the membrane are also labeled.

HD5 is a 32-residue peptide (Figure 1A) with a total charge of +4 at neutral pH. As with other defensins, HD5 contains three β-strands linked by one coil (T1) and a turn (T2) (Figure 1A,B). Various oligomeric states of HD5 have been found, but a dimer has been identified to be the most active form in solution [15,16,17]. Several studies have demonstrated that HD5 kills bacteria by disrupting the bacterial cell membrane via pore formation, and then penetrating into the bacterial cytoplasm [18,19,20]. The arginine-rich or active region is employed to adhere to the bacterial cell membrane. Electrostatic interactions between HD5 and the negatively charged lipopolysaccharide (LPS) surface are a major driving force for HD5 adhesion [21,22,23,24]. Although the adsorption mechanism of a dimeric HD5 on a membrane surface has recently been shown [21,22], the molecular-level understanding of how HD5 destabilizes the bacterial LPS membrane remains unclear. Thus, in this work, the dynamics of the HD5 dimer at five different positions (see Figure 2A for the starting positions) in a bacterial LPS membrane was investigated using molecular dynamics (MD) simulations. These five HD5 orientations were obtained from steered molecular dynamics (SMD) simulations. All HD5–membrane complexes were selected from positions that exert steered forces to pass through (see Figure S1 for force profiles). A simplified LPS membrane consisting of lipid A and 3-deoxy-d-manno-octulosonic acid (KDO) was used as a model membrane in this work (Figure 1C). Our results revealed that chain *b* interacts with the LPS membrane more than chain *a* does, implying that the two chains of an HD5 dimer perform different roles once the peptide is in a bacterial membrane. We speculate that this observation could explain why HD5 needs to be at least in dimeric form to be biologically functional. Taken together, this work provides more insights into how HD5 interacts with different regions of a bacterial membrane and demonstrates how this host-defense peptide disrupts the membrane, leading to leakage and cell death.

## 2. Results and Discussion

The starting and final orientations of the HD5 dimer are shown in Figure 2. Five different initial HD5 positions were simulations: (1) top of LPS membrane, (2) between the charged layer and hydrophobic tail of lipid A, (3) center of a membrane core, (4) phospholipid tails, and (5) phospholipid tail-phosphate (Pi) interface. The dimerization of HD5 remained intact at the end of 500 ns in all systems. The HD5 dimers at positions 1–3 were found to stably reside on the LPS surface, whereas the HD5 dimers at positions 4 and 5 remained inserted in the membrane (Figure 2A). Interestingly, a dimeric HD5 that started from the middle of the membrane (position 3) was eventually dragged up to the LPS surface. In contrast, the embedded HD5 peptides at positions 4 and 5 generated a water-filled pore throughout the duration of the simulations (Figure 2B). Moreover, a comparable membrane thickness was observed in all simulations, suggesting that the HD5 insertion does not have a significant effect on the membrane thickness.

**Figure 3:**
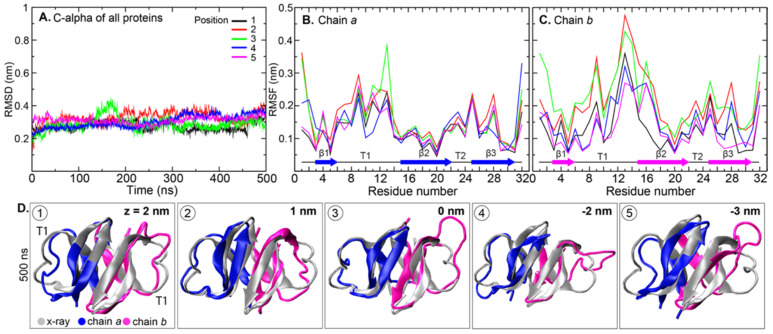
(**A**) Cα RMSDs of HD5 protein, (**B**) Cα RMSF of chain *a,* and (**C**) Cα RMSF of chain *b.* RMSD and RMSF were computed using the structure at t = 0 ns as the reference. (**D**) Superimposition of each snapshot at 500 ns and X-ray structure of HD5 (PDB ID: 1ZMP).

To examine structural flexibility, the Cα root-mean-square deviations (RMSDs) and fluctuations (RMSFs) were computed. RMSDs of HD5 in all systems ranged from 0.25 to 0.35 nm; slightly higher RMSDs were observed at positions 2, 4, and 5 (Figure 3A). Between the two chains, chain *b* was observed to be more flexible than chain *a* (Figure 3B,C). Furthermore, the RMSF data indicated large conformational fluctuations in the T1 region, especially T1 in chain *b* when HD5 was placed in positions 2 and 3 (Figure 3B,C). This observation indicates the conformational flexibility of T1. Highly flexible regions (residues 22–28) located on T2 and β2 were also observed. These regions harbor the active region. To elucidate how HD5 adopts its conformation once it is inside the bacterial membrane, the final orientation of the HD5 dimer in each simulation was superimposed on the X-ray structure of HD5 (PDB code: 1ZMP), as shown in Figure 3D (C-alpha RMSDs of these can be seen in Figure S2). The β-strand core was preserved in all systems, while the reorientation of T1 was visible (Figure 3D). This finding supported the high RMSFs observed in T1 regions in simulations. A larger T1 displacement was seen in chain *b*, especially in the case of embedded chain *b* at positions 3–5 (Figure 3D). The driving force for T1 reorientation is discussed later.

Looking at the hydrogen bonds formed by HD5 with the membrane and water molecules, it is clear that more HD5-membrane hydrogen bonds were observed when HD5 localized in the membrane core (~23–24 hydrogen bonds), while HD5 at the membrane–water interface formed approximately 10 hydrogen bonds (Table 1). Moreover, it is expected that water-exposed positions allow HD5 to be in contact with more water molecules (Table 1). Therefore, more water contacts were found at positions 1–3 (~130 hydrogen bonds). Despite the fact that chains *a* and *b* were identical, both chains seemed to show different degrees of binding affinities to the membrane. Chain *b* seemed to show more membrane contacts than chain *a*. The more interactions formed by chain *b* were due to the high flexibility of the T1 region (Figure 3C). Furthermore, the more membrane interactions formed by chain *b* suggested the importance of one chain to enable the adsorption of a dimeric HD5 on the LPS membrane. Chain *b* not only formed more membrane contacts but also showed more water exposure (Table 1). At positions 4 and 5, although HD5 dimers were inserted into a hydrophobic region, the HD5–water interactions reported in Table 1 confirmed the presence of a water-filled pore in the membrane core generated by HD5. Simulations extended to 1000 ns for positions 4 and 5 illustrated that the positions of HD5 and water-filled pores were well-preserved (Figure S3 in Supplementary Materials). This result showed the stability of a dimeric HD5 inside a hydrophobic core assisted by an aqueous environment.

**Figure 4:**
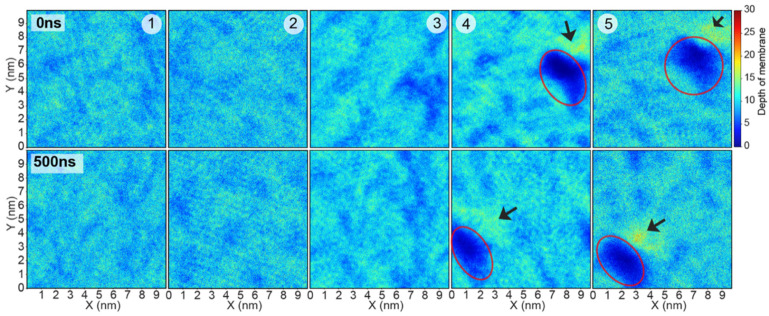
Density contour maps of LPS membrane at 0 ns and 500 ns for the five systems. Red ovals indicate the location of a dimeric HD5. Clack arrows represent the location of LPS accumulation.

Density maps of LPS membrane are displayed in Figure 4. Surface-lining HD5 dimers at positions 1 and 2 had no significant effect on LPS distribution throughout the course of the 500 ns simulations. For position 3, where the HD5 dimer was originally in between hydrophilic and hydrophobic regions, the movement of HD5 to the LPS surface smoothened the LPS surface, resulting in the channel filling (position 3 in Figure 2C and Figure 4). On the other hand, the membrane-embedded HD5 peptides at positions 4 and 5 maintained the water-filled channel (Figure 4). In addition, small blebs of LPS at the channel edge were observed (arrows in 4 and 5 in Figure 4). This was due to the accumulation of LPS in this region.

**Figure 5:**
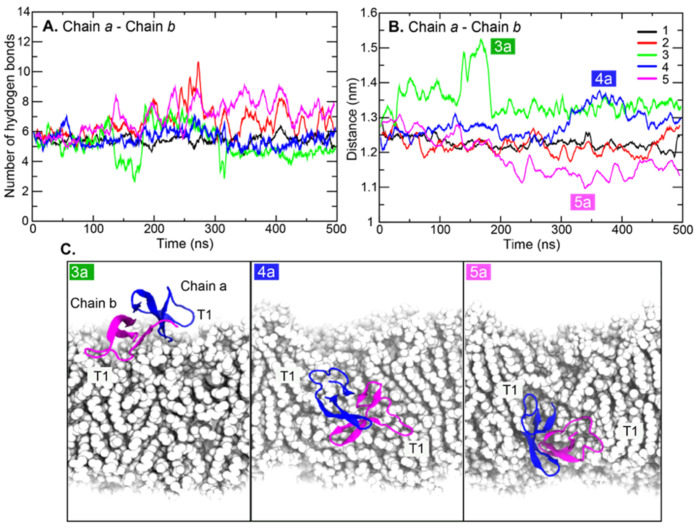
(**A**) Number of hydrogen bonds between chains *a* and *b* and (**B**) distance between the center of mass of chain *a* and chain *b* as a function of time. (**C**) HD5 orientations at positions 3a, 4a, and 5a. Chains *a* and *b* are colored in blue and magenta, respectively. T1 loop regions from each chain are also labeled.

The distances between the center of mass (COM) and hydrogen bonds between two chains were also calculated (Figure 5). Persistent hydrogen bonds between chains *a* and *b* indicated a stable dimer interface in simulations (Figure 5A). Altered chain *a-*chain *b* distances in Figure 5B demonstrated the flexibility of the dimeric HD5 interface. In Figure 5B, the large shifts in chain *a–*chain *b* distances from each position are labeled as 3a (position 3), 4a (position 4), and 5a (position 5). For 3a, the shifted distance occurred because the T1 regions were tethered by polar moieties of lipid A from the outer leaflet corresponding to the drop of hydrogen bonds, and this elongated the distances between the two chains (Figure 5), corresponding to the shift in radius of gyration in Figure S4. A similar scenario was also observed at position 4 (4a in Figure 5A and radius of gyration in Figure S4). T1 in chain *a* was trapped by polar lipid A head groups, while that of chain *b* became anchored by phospholipid head groups of the inner leaflet. Their orientation can be seen in Figure 5C. Nevertheless, the drop in distances in 5a indicated the tight packing of HD5 corresponding to a high number of chain *a*–chain *b* interactions (Figure 5A). Overall, the motion of T1 appeared to dominate the protein dynamics.

To investigate binding energetics, the membrane-HD5 binding energies were also calculated using the MM/PBSA method (Table 2). HD5 dimers employed electrostatic interactions to reside on/in the LPS membrane (attractive electrostatic energies of −45 to −52 kJ/mol), while hydrophobic forces did not make a major contribution. The HD5 dimer embedded at position 4 seemed to be the most stable conformation due to the most favorable total energy (~ −51 kJ/mol). HD5 at position 4 was stable as it was stabilized by polar moieties from both outer and inner leaflets. However, remaining on the LPS surface was also favored by HD5. Staying in the inner leaflet close to the phospholipid head groups appeared to be least favorable for the dimeric HD5 protein (Table 2). Insignificant solvation energies found in all cases confirmed the high impact of the hydrophilic-lining surfaces of the LPS membrane.

**Figure 6:**
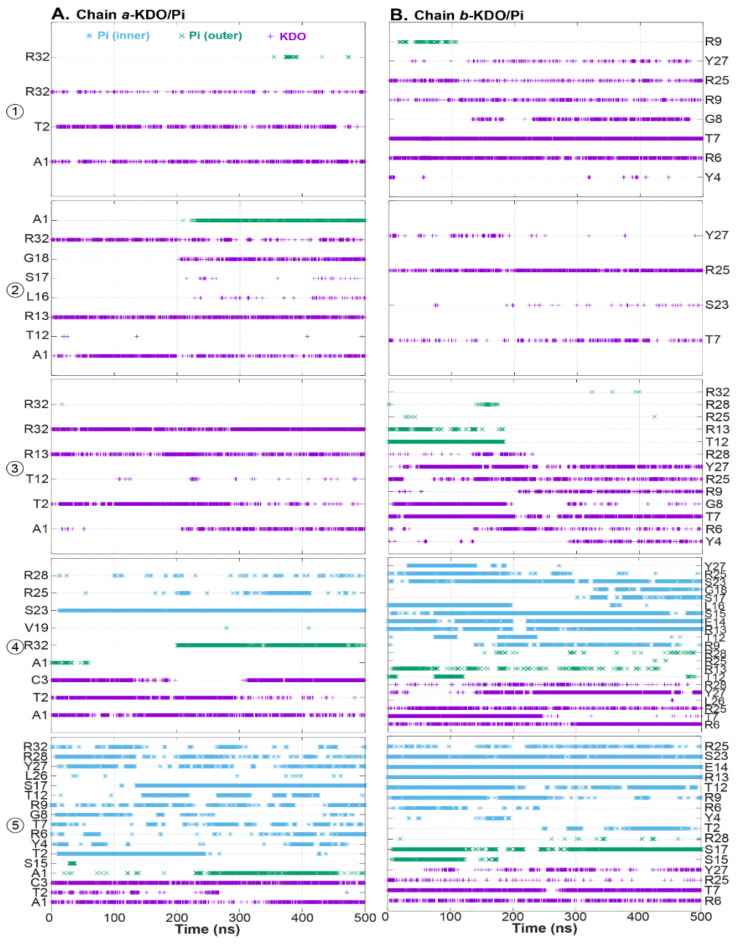
Hydrogen bonds formed between polar moieties of the membrane (KDO and Pi groups) and (**A**) chain *a* and (**B**) chain *b* of HD5. Interactions with KDO, Pi of lipid A (Pi(outer)), and Pi of phospholipid head groups (Pi(inner)) are colored in violet, green, and cyan, respectively.

**Figure 7:**
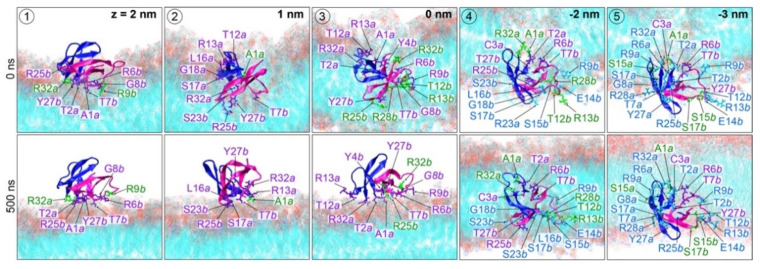
Orientation of dimeric HD5 molecules. Key residues that form hydrogen bonds in Figure 6 are labeled. Residues that interact with KDO, Pi of lipid A (Pi(outer)), and Pi of phospholipid head groups (Pi(inner)) are colored in violet, green, and cyan, respectively. The lowercase “*a*” and “*b*” letters indicate the chain of HD5.

To understand how HD5 interacts with an LPS membrane, hydrogen-bond analysis between residues and polar groups on a membrane was conducted. For the first three positions (positions 1–3), the final position of HD5 was at the LPS membrane surface. The interactions with KDO were the major driving forces that stabilized the dimeric HD5 at the membrane surface. Nonetheless, the phosphate group (Pi) became important when HD5 was inserted into the LPS membrane (Figure 6). Two HD5 orientations were captured on the LPS surface. A dimeric HD5 can align either in a near normal position (positions 1 and 3) or parallel (position 2) to the membrane axis (Figure 1B,C and Figure 7). For positions 1 and 3, chain *b* was the main player that adhered to the KDO layer involving interactions with Y4, R6, T7, G8, R9, R25, and Y27, whereas chain *a* employed A1, T2, R13, and R32 (Figure 6). In the case of position 2, a HD5 dimer sat on the membrane interface with interactions formed by A1, R13, G18, and R32 of chain *a*, while chain *b* contributed with T7 and R25 (Figure 6 and Figure 7). Arginine residues in the active region were involved in all surface-oriented HD5 cases. This observation highlighted the important role of the arginine residues in binding to the LPS membrane. Furthermore, at position 3, HD5 was initially located in the middle of the membrane and formed interactions with both KDO and Pi on an LPS surface (Figure 6). This position was stabilized by interactions from both chains *a* (R13 and R32) and *b* (T7, T12 (Pi), R13 (Pi), R25, Y27, and R28) (Figure 6). Nevertheless, the movement of HD5 to the water–membrane interface caused the disruption of interactions with T12, R13, and R28 and, at the same time, permitted KDO to interact with A1 (chain *a*), Y4 (chain *b*), R6 (chain *b*), and R9 (chain *b*) (Figure 6 andFigure 7). The formation of interactions with Y4, R6, and R9 with the T1 region of chain *b* along with A1 of chain *a* dragged HD5 up to the membrane–water interface (Figure 6), suggesting that electrostatic interactions between HD5 and lipid A head groups (KDO and Pi(outer)) were the main barrier for HD5 insertion.

In the case of HD5 at positions 4 and 5, both HD5 dimers aligned perpendicular to the membrane axis (Figure 7), but HD5 at position 5 appeared to slightly tilt away from its axis. This tilted pose was due to its interaction with Pi(inner) (Figure 7). HD5 dimers at positions 4 and 5 interacted with polar groups of both inner and outer leaflets. As with the other HD5 simulations, chain *b* at position 4 was the main contributor of membrane contacts. Its T1 region (residues 6–14) formed strong interactions with Pi(inner), whereas β2 and a part of β3 assisted with this interaction network (R9, T12, R13, E14, S15, L16, S17, G18, S23, R25, and Y27). The β3 region also contributed to the contacts with KDO and Pi(outer) (R6, T7, R25, Y27, and R28). Moreover, chain *a* interacted with polar moieties from both leaflets using parts of β2 and β3. The inserted HD5 dimers at positions 4 and 5 were stabilized by polar moieties from both membrane leaflets. This was because the passage of HD5 dragged lipid A head groups down into the membrane core, resulting in the water-filled pore, as shown in Figure 2.

For position 5, the HD5 dimer was originally located at the water–inner leaflet interface. During the course of the 500 ns simulation, HD5 was pushed into the inner leaflet, resulting in a lower water exposure (Figure 2 and Table 1). Inside the membrane, dimeric HD5 was stabilized mainly by Pi(inner), but some interactions with KDO and Pi(outer) were also observed. As reported earlier, the interactions with KDO and Pi(outer) were due to HD5 translocation that pulled some lipid A head groups down with it. With the inner leaflet, both chains showed comparable degrees of membrane-binding affinities. Both chains employed residues on T1 (R9, T12, R13, and E14) and part of the β3 regions (Y27, R28, and R32 for chain *a* and S23 and R25 for chain *b*) to contact with Pi(inner) (Figure 6). HD5 was dragged back into the hydrophobic core by strong electrostatic forces involving KDO and Pi(outer), especially KDO. The water channel remained. The presence of the HD5-LPS head group interactions related to the accumulation of KDO and Pi(outer) inside a water-filled pore. This finding also reflected the role of LPS head groups (KDO and Pi(outer)) in the formation of the water channel.

Additionally, comparing positions 3, 4, and 5, interactions with Pi(inner) contributed to the stability of the membrane-spanning orientation of HD5 that also led to the formation of a water-filled pore. At position 3, no contact with Pi(inner) was observed, resulting in the expulsion of HD5 to the LPS surface. As reported earlier, KDO and Pi(outer) of the LPS layer facilitated HD5 adsorption, and they simultaneously acted as retarders for HD5 penetration. Insertion was accomplished with the assistance of Pi(inner) from the inner leaflet. In addition, this work demonstrates that a water-filled pore could be formed by a single HD5 dimer. The membrane-spanning conformation of HD5 was stable enough to generate the water-filled hole. At position 5, HD5 was pushed into the membrane core due to the persistent drag of the polar moieties in the membrane. The electrostatic interactions appeared to play a role in retarding the release of HD5 to the interior.

Although permeation was not observed here, our results demonstrate water leakage caused by the membrane-spanning dimeric HD5, as reported in other defensins such as HNP-2 and HNP-3 [25]. An HD5 dimer appeared to employ a similar membrane-embedding mechanism to HNP-3, where the hydrophobic region of HD5 interacts with the hydrophobic membrane core while leaving the active region in contact with the charged headgroup [26]. Membrane penetration and pore formation are commonly employed by defensins to inactivate bacteria. However, the antimicrobial activity of HNP-3 was also reported to be due to the reduced expression of microbial ligand receptor (CD98) on human epithelial cells, resulting in the prevention of bacterial invasion [27]. It is also worth exploring the possibility of HD5-retarding bacterial invasion as performed by HNP-3.

## 3. Materials and Methods

### 3.1. HD5–Membrane Complex Setup

The starting HD5–LPS complexes here were obtained from our previous work [21]. The final snapshots of the dimeric HD5–LPS complex obtained were used here. A simplified LPS model (the inner core region (Re-LPS) composed of lipid A and KDO) was employed using LPS parameters reported previously [28]. Constant-velocity SMD simulations were performed for 200 ns. An external force was applied in the z-direction (perpendicular to the membrane axis) by attaching a virtual harmonic spring with a force constant of 1000 kJ mol^−1^ nm^−2^ to the center of mass of a dimeric HD5 protein. The direction of pulling was from the outer leaflet to the inner leaflet (z→−z direction) at the rate of 0.04 nm/ns. Five positions (position 1 to 5) along the z direction were selected based on the exerted forces (at z = 2, 1, 0, −2, and −3 nm in Figure S1) to study the alternation of HD5 and membrane in comparison. The cartoon views of the initial positions of HD5 in the membrane are shown in Figure 2A and Figure S1. The equilibration runs were performed on all five HD5-LPS structures for 10 ns and were followed by the 500 ns production runs. At positions 4 and 5, the extended simulations to 1000 ns were conducted to further observe the membrane-spanning conformation of HD5 structures.

### 3.2. Molecular Dynamics Simulations

MD simulations were performed using the GROMACS 5.0 package with GROMOS 53A6 force fields [29]. The particle mesh Ewald (PME) method was used for electrostatic treatment with a short-range cut-off of 1 nm and a Fourier spacing of 0.12 nm. A 2 fs integration time step was used with LINCS algorithms. The simulations were conducted in the NPT ensemble using the semi-isotropic Parrinello–Rahman barostat with τ_p_ = 1 ps and a v-rescale thermostat at 323 K [22] with a coupling constant of τ_p_ = 0.1 ps, where protein, membrane, and solution were coupled separately.

All simulations were analyzed with GROMACS tools and in-house scripts. Hydrogen bonds were computed using gmx_hbond with default parameters (the hydrogen-donor–acceptor cutoff angle was set as 30° and the cutoff radius (X-acceptor) was set as 0.35 nm). Virtual molecular dynamics (VMD 1.9.3) was used for visualization and preparation of molecular graphic images [30]. 

### 3.3. MM/PBSA Calculations

The molecular mechanics Poisson–Boltzmann surface area (MM/PBSA) method was employed to calculate the binding free energy of the association of the protein–membrane complex. MM/PBSA models were generated using *g mmpbsa* by first PBC-correcting the MD trajectories. This method assumes that the protein and membrane conformations in the bound and unbound states are identical. A stable simulation trajectory between 300 ns and 500 ns of each position was chosen to calculate the MM/PBSA-based binding free energy.

## 4. Conclusions

In this work, the behavior of a dimeric HD5 along the membrane axis was investigated. The two chains of HD5 were found to contribute unequally to membrane adsorption and insertion. Our work demonstrated that one chain appears to play a key role in interacting with the LPS membrane. This not only agrees well with a previous study that showed that HD5 binds strongly to a lipid A moiety [19], but also highlights the synergetic roles of LPS head groups (KDO and Pi(outer)) in facilitating the adsorption and prevention of HD5 permeation. Strong hydrogen bonds between active region arginines and the sugar moieties of KDO facilitate spontaneous HD5 adsorption. When HD5 dives down into the membrane core, the strong interactions with the arginine drag lipid A head groups into the membrane core, resulting in the formation of a water-filled channel. Furthermore, this work also highlights the role of the T1 region in interacting with a membrane. The highly flexible T1 can grasp polar moieties from both leaflets, allowing HD5 to span across the membrane. Although multiple bacteria-killing mechanisms of HD5 have been reported [18,31], only pore-formation was observed in this work. A single dimeric HD5 seemed to form a water-filled pore. Neither membrane bleb nor severe LPS disruption were noted, suggesting that a higher HD5 concentration may be required. The movement of HD5 up to the membrane surface induced by LPS head groups (as seen at positions 2 and 3) suggests that the interaction with LPS is crucial. Strong attractive forces between cationic HD5 and LPS polar moieties suggest the primary role of HD5 in neutralizing the LPS surface. Thus, the LPS structure appears to play a key role in HD5 efficiency. Additionally, full-length LPS, including the O-antigen region, may provide further insights into how HD5 recognizes the microbial membrane and exhibits its function.

The findings from MD simulations reported here explain the membrane spanning mechanism of HD5, which could be useful for the design and development of HD5-analog drugs. However, no permeation was observed here, as such events occur at the timescale of minutes, which is generally not possible with most conventional MD simulations. Thus, advanced sampling techniques such as accelerated MD may be necessary to computationally demonstrate the penetration mechanism of HD5.

## Figures and Tables

**Table 1:** Average number of hydrogen bonds formed by HD5 protein with membrane and water molecules. Hydrogen bonds of each chain are also shown.

Position	Membrane			Water		
	Protein	Chain *a*	Chain *b*	Protein	Chain *a*	Chain *b*
1	10.82 ± 3.54	1.97 ± 1.34	8.85 ± 3.28	134.16 ± 7.39	70.30 ± 5.45	63.86 ± 5.61
2	9.71 ± 2.41	6.18 ± 1.93	3.54 ± 1.80	137.60 ± 8.29	62.98 ± 5.65	74.62 ± 5.85
3	14.08 ± 4.90	5.29 ± 1.86	8.56 ± 4.69	130.99 ± 10.48	63.71 ± 5.92	67.28 ± 9.16
4	23.99± 3.44	9.05 ± 1.94	14.95 ± 2.97	103.01 ± 8.13	47.32 ± 4.69	55.70 ± 6.36
5	23.33 ± 3.84	9.30 ± 2.46	14.03 ± 2.96	99.48 ± 8.26	44.70 ± 5.40	54.78 ± 6.04

**Table 2:** Free energy of binding of protein and membrane at the five positions.

Binding Energy of HD5-Membrane (×10^3^ kJ/mol)	Position				
	1	2	3	4	5
∆E_vdW_	−0.06 ± 0.01	−0.21 ± 0.02	−0.15 ± 0.02	−1.03 ± 0.06	−0.42 ± 0.04
∆E_Elec_	−45.74 ± 1.15	−51.50 ± 1.12	−49.30 ± 0.95	−52.72 ± 0.47	−41.80 ± 0.60
∆E_polar solv_	1.02 ± 0.39	2.00 ± 0.39	0.28 ± 0.42	2.65 ± 0.37	2.40 ± 0.69
∆E_non-polar solv_	0.25 ± 0.03	0.23 ± 0.02	0.24 ± 0.01	0.14 ± 0.03	0.19 ± 0.02
∆G_total_	−44.54 ± 1.11	−49.49 ± 0.82	−48.93 ± 0.76	−50.97 ± 0.57	−39.63 ± 1.03

## Supplementary Materials

The following are available online at https://www.mdpi.com/article/10.3390/ijms222212401/s1.
