# Supplementary material for: Evaluation of the Binding Mechanism of Human Defensin 5 in a Bacterial Membrane: A Simulation Study
Source: Int J Mol Sci. 2021 Nov 17;22(22):12401. doi: 10.3390/ijms222212401 (PMC8619297; doi:10.3390/ijms222212401)
Supplement: Supplementary file 1 [file ijms-22-12401-s001.zip › ijms-1462102-supplementary.pdf]

Evaluation of the binding mechanism of human defensin 5 in a bacterial membrane: A simulation study

Tadsanee Awang[1], Phoom Chairatana[2], Ranjit Vijayan[3,4,5,*], and Prapasiri Pongprayoon[1,6,*]

[1]Department of Chemistry, Faculty of Science, Kasetsart University, Chatuchak, Bangkok, 10900, Thailand.

[2]Department of Microbiology, Faculty of Medicine Siriraj Hospital, Mahidol University, Bangkok, 10700, Thailand

[3]Department of Biology, College of Science, United Arab Emirates University, PO Box 15551, Al Ain, United Arab Emirates

[4]Big Data Analytics Center, United Arab Emirates University, PO Box 15551, Al Ain, United Arab Emirates

[5]Zayed Center for Health Sciences, United Arab Emirates University, PO Box 17666, Al Ain, United Arab Emirates

[6]Center for Advanced Studies in Nanotechnology for Chemical, Food and Agricultural Industries, KU Institute for Advanced Studies, Kasetsart University, Bangkok 10900, Thailand.

*To whom correspondence should be addressed:

E-mail: ranjit.v@uaeu.ac.ae

Telephone: +971-3-713-6302

Fax: +971-3-713-6944

E-mail: prapasiri.p@ku.th

Telephone: +66-2562-5555

Fax: +66-2579-3955

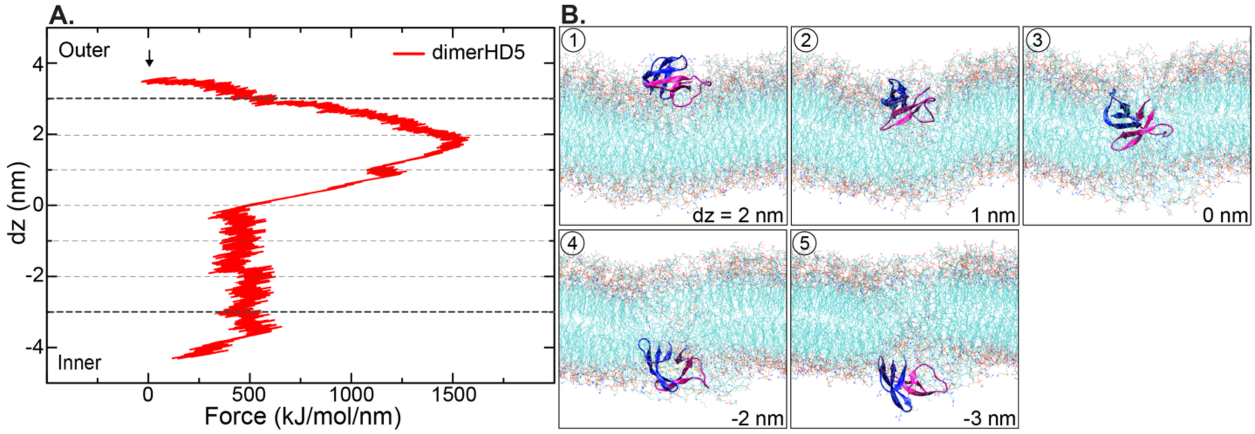

Figure S1. (A) Pull force profiles from constant velocity SMD simulations of HD5 through LPS membrane. (B) The initial snapshot at 5 positions, corresponding to the z-axis position of 2 nm, 1 nm, 0 nm, -2 nm, and -3 nm. Blue and magenta color represents chain *a* and chain *b* of HD5.

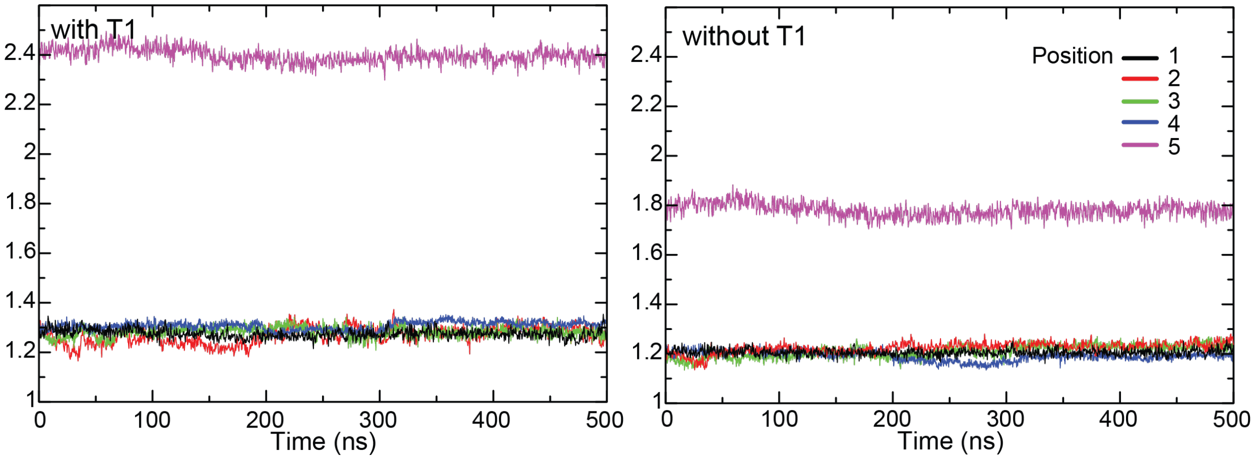

Figure S2. C-alpha RMSDs of all system when the X-ray structure (PDB code: 1ZMP) is used as a reference structure.

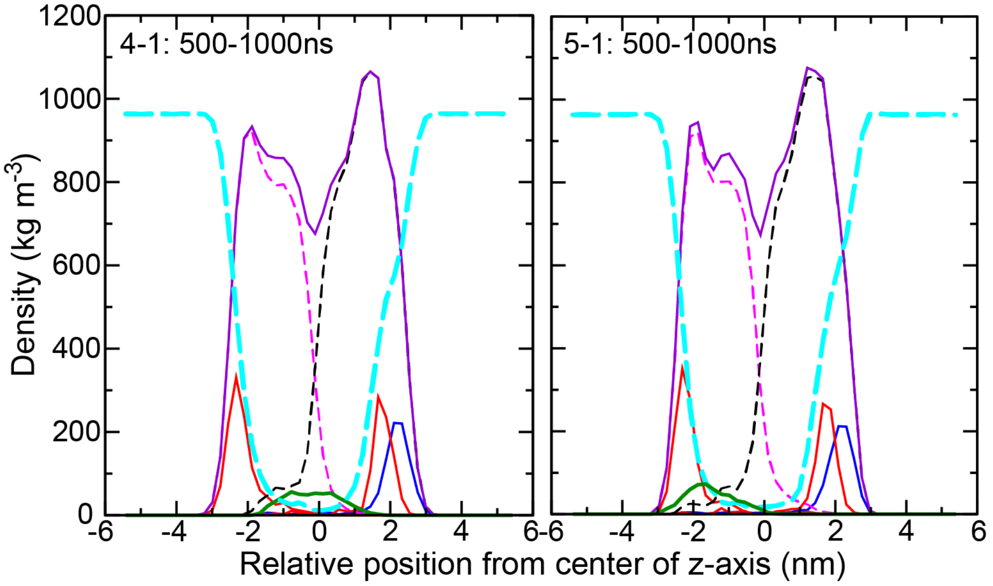

Figure S3. Density profiles of key components from extended 1000 ns simulations. The subgroups are dimeric HD5 (green), membrane (purple), outer leaflet (black dashed line), inner leaflet (magenta dashed line), KDO (blue), Pi (red), and water (cyan dashed line).

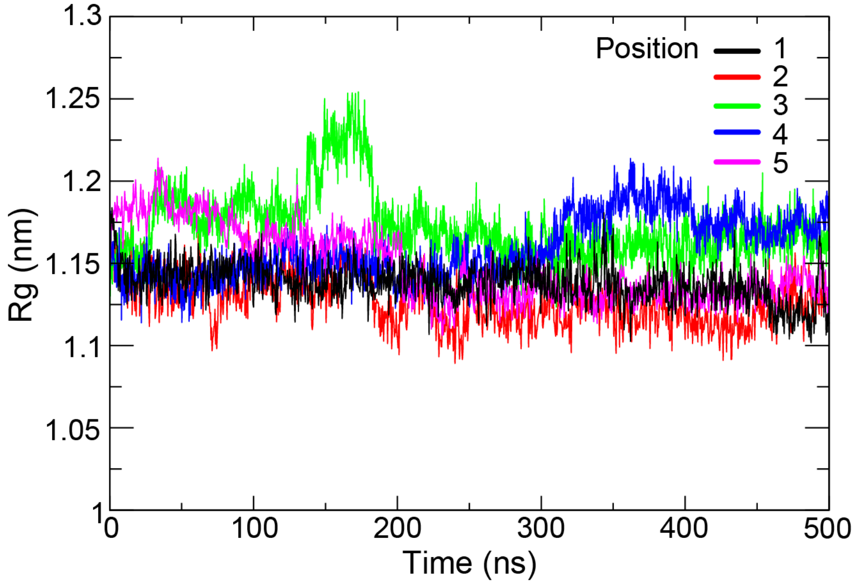

Figure S4. Radii of gyration of HD5 in all systems
